# RXR Ligands Negatively Regulate Thrombosis and Hemostasis

**DOI:** 10.1161/ATVBAHA.117.309207

**Published:** 2017-03-02

**Authors:** Amanda J. Unsworth, Gagan D. Flora, Parvathy Sasikumar, Alexander P. Bye, Tanya Sage, Neline Kriek, Marilena Crescente, Jonathan M. Gibbins

**Affiliations:** From the Institute for Cardiovascular and Metabolic Research, School of Biological Sciences, University of Reading, United Kingdom.

**Keywords:** atherosclerosis, eczema, platelet activation, thrombin

## Abstract

**Objective—:**

Platelets have been found to express intracellular nuclear receptors including the retinoid X receptors (RXRα and RXRβ). Treatment of platelets with ligands of RXR has been shown to inhibit platelet responses to ADP and thromboxane A2; however, the effects on responses to other platelet agonists and the underlying mechanism have not been fully characterized.

**Approach and Results—:**

The effect of 9-*cis*-retinoic acid, docosahexaenoic acid and methoprene acid on collagen receptor (glycoprotein VI [GPVI]) agonists and thrombin-stimulated platelet function; including aggregation, granule secretion, integrin activation, calcium mobilization, integrin αIIbβ3 outside-in signaling and thrombus formation in vitro and in vivo were determined. Treatment of platelets with RXR ligands resulted in attenuation of platelet functional responses after stimulation by GPVI agonists or thrombin and inhibition of integrin αIIbβ3 outside-in signaling. Treatment with 9-*cis*-retinoic acid caused inhibition of thrombus formation in vitro and an impairment of thrombosis and hemostasis in vivo. Both RXR ligands stimulated protein kinase A activation, measured by VASP S157 phosphorylation, that was found to be dependent on both cAMP and nuclear factor κ-light-chain-enhancer of activated B cell activity.

**Conclusions—:**

This study identifies a widespread, negative regulatory role for RXR in the regulation of platelet functional responses and thrombus formation and describes novel events that lead to the upregulation of protein kinase A, a known negative regulator of many aspects of platelet function. This mechanism may offer a possible explanation for the cardioprotective effects described in vivo after treatment with RXR ligands.

## Abstract 2

Supplemental Digital Content is available in the text.

Many intracellular nuclear receptors are expressed in human platelets that negatively regulate platelet function when stimulated by their endogenous ligands.^1–12^ Retinoid X receptors (RXR) (α, β, and γ) regulate transcription and expression of specific genes involved in cell proliferation, differentiation, and lipid metabolism and are activated by retinoids and vitamin A derivatives.^13–17^ RXR ligands have been found to exert cardioprotective effects by reducing atherosclerosis in apolipoprotein E knockout mice,^18^ and treatment with RXR agonists has also been shown to sensitize cells to insulin and rescue hyperglycemia and hyperinsulinemia in type 2 diabetes mellitus mouse models, highlighting their potential role as therapeutics for the treatment of type 2 diabetes mellitus.^19^

Several recent studies have identified acute, nongenomic roles for several members of the nuclear hormone superfamily including RXR.^1,3–5,11,20,21^ Treatment with nuclear receptor ligands is associated with cardioprotective effects, whereas ligands for RXR are associated with reduced atherosclerosis and inflammation,^18^ which may be because of regulation of platelet activity. 9-*cis*-retinoic acid (9-*cis*-RA), which is marketed as Alitretinoin, is used for the treatment of Kaposi sarcoma and chronic hand eczema, and decreased blood clotting is listed as one of its side effects.^22,23^

Treatment of platelets with RXR ligands has been shown to inhibit ADP and thromboxane A2 receptor–induced platelet activation via inhibition of Gq.^5^ However, effects of RXR ligands on platelet responses to other receptor agonists (such as thrombin and low concentrations of collagen and other glycoprotein VI [GPVI] receptor agonists) and integrin α_IIb_β_3_ function are unknown, and the antithrombotic effects of RXR agonists have not be studied.

This study set out to investigate the mechanism by which RXR ligands regulate platelet function and thrombus formation and explore the potential antiplatelet properties of RXR ligands. We describe that RXR agonists evoke broad inhibition of platelet function, resulting in inhibition of thrombus formation in vitro and in vivo. We report that RXR ligands increase protein kinase A (PKA) activity via both cAMP and nuclear factor κ-light-chain-enhancer of activated B cells (NFκB)–dependent mechanisms that have not been described for the endogenous agonist of any other nuclear receptor.^24^

## Materials and Methods

Materials and Methods are available in the online-only Data Supplement.

## Results

### Expression and Localization of RXR in Platelets

**Figure 1.**
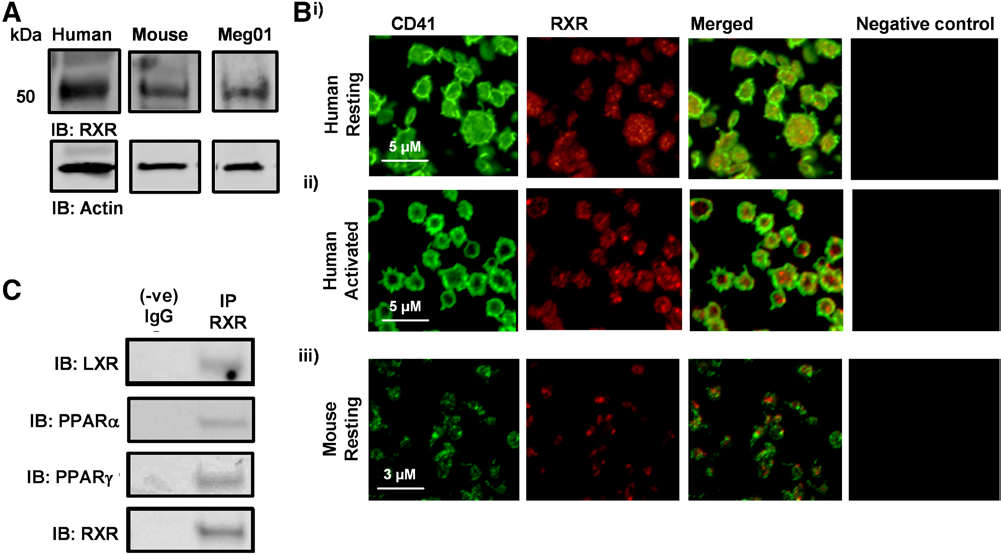
Expression and localization of retinoid X receptors (RXR) in platelets. Human and mouse washed platelets were (**A**) lysed in (SDS–PAGE) Laemmli sample buffer, separated by SDS–PAGE and transferred to polyvinylidene fluoride (PVDF) membranes before blotting with an antibody that recognizes both α and β isoforms of RXR. Representative blots are shown. **B**, Human platelets ([i] resting and [ii] activated) and (iii) mouse platelets (resting) were fixed in 4% paraformaldehyde and permeabilized with 0.2% Triton-X-100 and stained for RXR (in red) and CD41 (in green, as a marker for the platelet membrane) with primary antibodies raised against RXR and CD41. Secondary antibodies conjugated to Alexa-647 and Alexa-488 were used to visualize RXR and CD41, respectively. No primary antibody–treated samples were also included as a negative control. Representative images shown. **C**, Human washed platelets lysed in NP40 buffer before immunoprecipitation of RXR using 1 μg/mL of primary antibody overnight at 4°C and protein A/G magnetic beads. Pull down samples lysed in (SDS–PAGE) Laemmli sample buffer, separated by SDS–PAGE, and transferred to PVDF membranes before blotting with antibodies that recognize different nuclear receptors, including PPARα, PPARγ, and LXR (liver X receptor) and a secondary antibody that does not recognize denatured IgG. RXR IgG loaded as a control for IgG contamination. Representative blot shown.

Here, we confirm protein expression of RXR in both human and mouse platelets and Meg01 cells using immunoblot analysis (Figure 1A) and also immunohistochemistry (Figure 1B and 1C). This is further supported by previously described expression of the separate RXR isoforms in both the human and mouse transcriptome and protein expression in human platelets.^5,25^ Staining of platelets with antibodies raised against both RXR isoforms and the membrane marker CD41 confirmed the presence of RXR in both human and mouse platelets with punctate staining throughout the cytosol within resting human platelets, which was not altered on platelet activation. Coimmunoprecipitation experiments were performed to determine whether RXR could form heterodimers with other nuclear receptors in platelets (as in other cell types). RXR was found to coimmunoprecipate with the peroxisome proliferator–activated receptors (PPARs), PPARα, PPARβ, and PPARγ, and LXR (liver X receptor; Figure 1C), thereby suggesting that RXR can form heterodimers with other nuclear receptors in human platelets.

### RXR Agonists Inhibit Platelet Aggregation to a Range of Agonists

**Figure 2.**
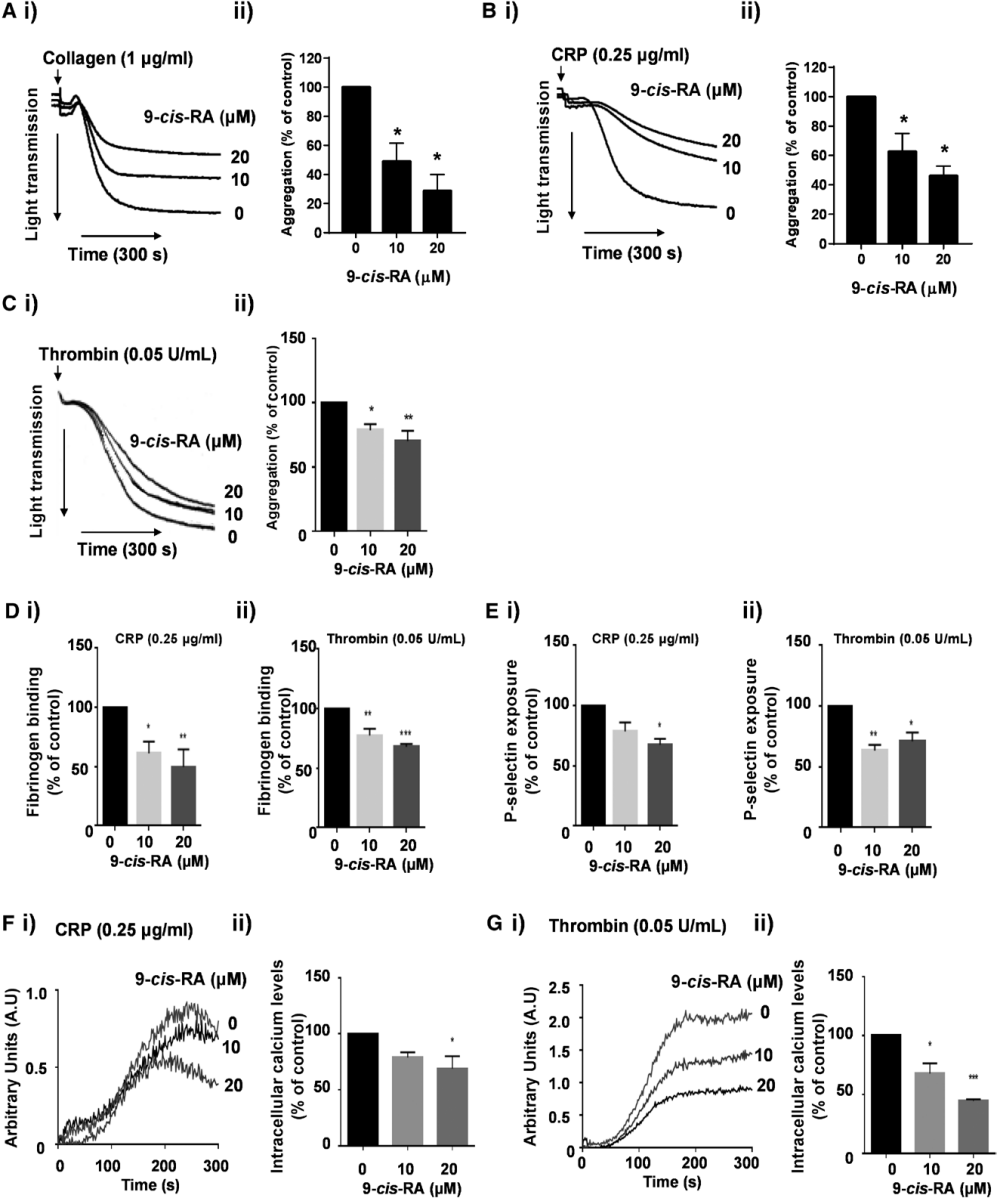
The effect of retinoid X receptors (RXR) ligands on platelet function. Washed human platelets were pretreated for 10 min with increasing concentrations of 9-*cis*-RA (10 and 20 μmol/L) before stimulation with (**A**) collagen (1 μg/mL), (**B**) CRP-XL (0.25 μg/mL), or (**C**) thrombin (0.05 U/mL) and aggregation monitored using optical light transmission aggregometry, (i) representative traces and (ii) quantified data shown. **D**, Integrin activation measured as fibrinogen binding in (i) CRP-XL–stimulated and (ii) thrombin-stimulated platelets. **E**, α-Granule secretion measured as P-selectin exposure in (i) CRP-XL–stimulated and (ii) thrombin-stimulated platelets. Intracellular calcium levels determined in FURA-2 AM–loaded platelets after stimulation with (**F**) CRP-XL (0.25 μg/mL) and (**G**) thrombin (0.05 U/mL); (i) representative traces and (ii) quantified data shown. Data expressed as the percentage of untreated control. Results are mean±SEM for n≥3. *P≤0.05 in comparison to vehicle controls.

Light transmission aggregometery using human washed platelets was used to analyze the effects of the endogenous RXR ligands 9-*cis*-RA, docosahexaenoic acid, and synthetic ligand methoprene acid on platelet aggregation. We found that 9-*cis*-RA, docosahexaenoic acid, and methoprene acid at the concentrations used (10 and 20 μmol/L) did not cause platelet aggregation in the absence of platelet agonists (Figure IA in the online-only Data Supplement). As shown in Figure 2, pretreatment with 9-*cis*-RA (20 μmol/L) inhibited platelet aggregation to collagen (1 μg/mL; Figure 2A), the GPVI collagen receptor–specific agonist, CRP-XL (0.25 μg/mL; Figure 2B), and thrombin (0.05 U/mL; Figure 2C) compared with vehicle controls (containing 0.1% dimethyl sulfoxide), with ≈60% and 20% inhibition after stimulation by collagen (and CRP-XL) and thrombin, respectively. Treatment with docosahexaenoic acid or methoprene acid (10 or 20 μmol/L) also inhibited aggregation to collagen and thrombin (Figure IB through IE in the online-only Data Supplement). As collagen-induced platelet aggregation is partially dependent on the release of secondary mediators and RXR agonists have been shown to inhibit platelet responses to ADP and thromboxane A2,^5^ we determined whether the inhibitory effects of the RXR agonists were because of their ability to inhibit secondary mediator signaling. The effects of 9-*cis*-RA on collagen-evoked aggregation were found to be additive to the inhibition caused by blockade of secondary mediator signaling and suggested that 9-*cis*-RA was able to inhibit collagen-evoked signaling directly (Figure IF in the online-only Data Supplement).

Currently available antagonists for RXR, including HX531, are classified by their ability to inhibit the genomic functions of the receptor, and it is unknown how they affect the nongenomic actions of RXR. End point aggregation assays were performed in 96-well microtiter plates in the presence or absence of HX531 (10 and 30 μmol/L) to a range of platelet agonist concentrations (Figure IG in the online-only Data Supplement). Interestingly, HX531 caused inhibition of platelet aggregation after stimulation by collagen, thrombin, and the thromboxane A2 receptor agonist U46619, which is similar to the inhibition observed previously by 9-*cis*-RA, docosahexaenoic acid, and methoprene acid. HX531 binds to the same binding pocket in the RXR receptor as the 2 RXR agonists 9-*cis*-RA and methoprene acid. Although HX531 functions to block the DNA-binding ability of the RXR receptor, it is possible that alteration of the DNA-binding region is not involved in the nongenomic functions of this receptor, whereas other conformational changes that occur after ligand interaction with the binding pocket may be involved. These findings suggest that HX531 acts as an agonist and not an antagonist of the nongenomic functions of RXR.

### RXR Agonists Reduce Integrin Activation and α-Granule Secretion

Platelet inside-out signaling is essential for activation of integrin α_IIb_β_3_ which supports aggregation by enabling integrin binding to fibrinogen and von Willebrand factor. Secretion of platelet granule contents amplifies platelet activation through recruitment of surrounding platelets, enhancing thrombus formation. To determine whether RXR ligands alter the activation of integrin α_IIb_β_3_ and α-granule secretion, fibrinogen binding and P-selectin exposure (a marker of α-granule secretion) on platelets pretreated with RXR ligands were determined in CRP-XL (0.25 μg/mL) and thrombin (0.05 U/mL)–stimulated platelets (Figure 2D and 2E). Consistent with the inhibition of platelet aggregation, fibrinogen binding and P-selectin exposure to both agonists was inhibited in 9-*cis*-RA–treated platelets compared with controls. Maximum inhibition of both fibrinogen binding (50% CRP-XL and 25% thrombin) and P-selectin exposure (20%–25% to either agonist) observed at the highest concentration of 9-*cis*-RA (20 μmol/L; Figure 2D and 2E). Similar results were obtained after treatment with methoprene acid (Figure IIA and IIB in the online-only Data Supplement).

### 9-*cis*-RA Inhibits Elevation of Intracellular Calcium

Elevation of cytosolic Ca^2+^ levels is a critical event in platelet activation and is stimulated by both collagen and thrombin, although via distinct mechanisms.^26^ As shown in Figure 2F and 2G, treatment with 9-*cis*-RA resulted in a reduction in peak cytosolic calcium levels of 25% after stimulation with CRP-XL (20 μmol/L 9-*cis*-RA) and ≈50% after thrombin stimulation in comparison to vehicle control. Treatment with methoprene acid had similar effects (Figure IIC and IID in the online-only Data Supplement). These observations indicate that RXR agonists are able to inhibit Ca^2+^ elevation evoked by both of these platelet agonists, despite the distinct signaling pathways that are activated by them.

### RXR Ligands Negatively Regulate Outside-In Signaling

**Figure 3.**
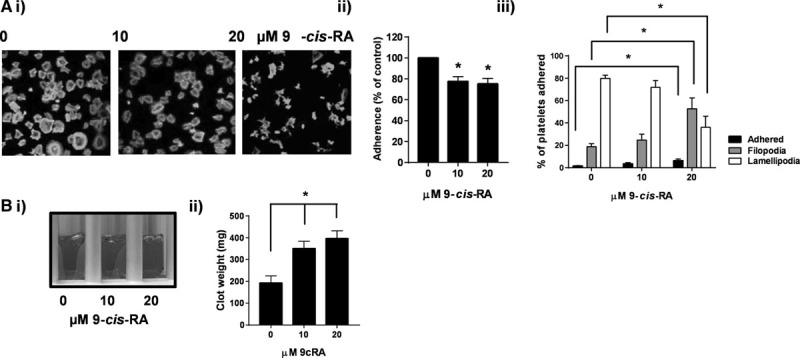
The effect of 9-*cis*-retinoic acid (9-*cis*-RA) on integrin αIIbβ3 outside-in signaling. Human washed platelets pretreated for 10 min with increasing concentrations of 9-*cis*-RA (10 and 20 μmol/L) or vehicle control were exposed to fibrinogen (100 μg/mL) coated coverslips. **A** (i) representative images of spreading and adhesion after 45 min. Platelets were stained with phalloidin Alexa-488 for visualization. Images were taken under oil immersion with magnification ×100. (ii) Adhesion: the number of platelets adhered were counted in 5 randomly selected fields of view, and the number of cells adhered were expressed as the percentage of the vehicle-treated control. (iii) Spreading platelets were classified into 3 different categories to determine the extent of their spreading (adhered but not spread; filopodia: platelets in the process of extending filopodia and lamellipodia: platelets in the process of extending lamellipodia including those fully spread). Results expressed (as relative frequency) as the percentage of the total number of platelets adhered. **B**, Clot retraction: human washed platelets pretreated for 10 min with increasing concentrations of 9-*cis*-RA (10 and 20 μmol/L) or vehicle control were added to aggregometer tubes in the presence of 2 mg/mL fibrinogen and 2 mmol/L CaCl_2_. Clot retraction was initiated by addition of thrombin 1 U/mL (final concentration) and left to proceed for 1 h at room temperature. Extent of clot retraction was determined by comparing clot weight. (i) Representative images using red blood cell stained platelet-rich plasma, (ii) data expressed as clot weight (mg). Unless stated otherwise, results are mean±SEM for n≥3. *P≤0.05 in comparison to vehicle controls.

Given that RXR agonists have broader inhibitory effects than initially appreciated, the effect of the RXR agonists on adhesion to fibrinogen and outside-in signaling evoked by integrin αIIbβ3 was also investigated. After treatment with 9-*cis*-RA (10 and 20 μmol/L), both adhesion and spreading on fibrinogen-(100 μg/mL)–coated coverslips were found to be inhibited in 9-*cis*-RA–treated samples compared with vehicle-treated controls (Figure 3A). Similar effects on adhesion and spreading on fibrinogen were observed after treatment with docosahexaenoic acid (Figure IIIA in the online-only Data Supplement).

Outside-in signaling is also essential for the regulation of clot retraction, which is required for thrombus stabilization. Consistent with the inhibition of adhesion and spreading on fibrinogen, treatment of platelets with 9-*cis*-RA resulted in an inhibition of clot retraction because clot weight was increased after treatment with 9-*cis*-RA compared with vehicle-treated controls (Figure 3B). Similar observations for docosahexaenoic acid and methoprene acid support the notion that the effect on clot retraction is mediated by RXR activity (Figure IIIB in the online-only Data Supplement).

### RXR Ligands Inhibit Thrombus Formation on Collagen Under Flow

**Figure 4.**
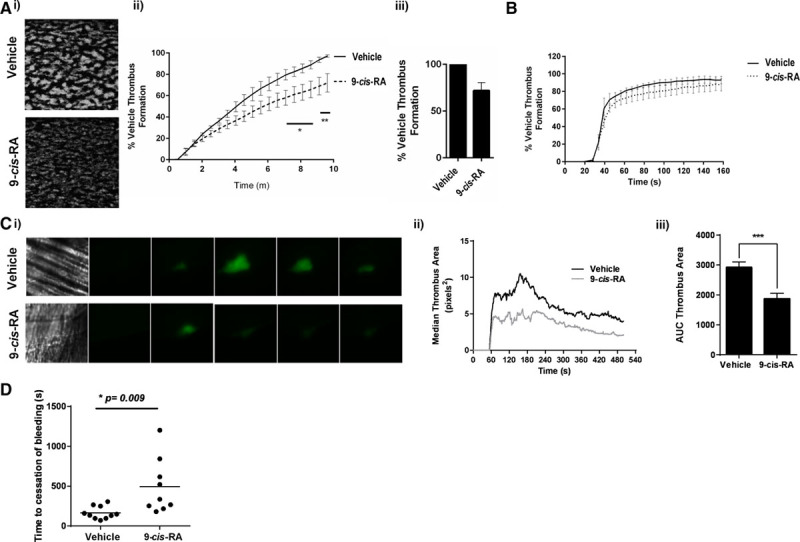
The effect of retinoid X receptor (RXR) ligands on thrombus formation in vitro and in vivo. **A** and **B**, DiOC6-loaded human whole blood was pretreated with vehicle (black) or 20 μmol/L 9-*cis*-retinoic acid (9-*cis*-RA; dotted) in the absence (**A**) or presence (**B**) of 10 μmol/L integrilin for 10 min before perfusion through collagen-coated (100 μg/mL) Vena8Biochips at an arterial shear rate of 20 dyn/cm^2^. Thrombus formation was determined after 10 min (**A**) or 3 min (**B**) by comparing fluorescence intensity in the vehicle- and 9-*cis*-RA–treated samples. (ii) Data expressed as percentage of vehicle control, where maximum fluorescence observed in vehicle-treated platelets is considered to be 100% thrombus formation. (iii) Data expressed as % of vehicle control after 10-min thrombus formation. **C**, In vivo thrombosis was assayed by intravital microscopy using the laser-induced injury model. 9-*cis*-RA (estimated concentration 20 μmol/L) or vehicle (0.1% v/v dimethyl sulfoxide) was administered intravenously to mice, and platelets were fluorescently labeled with Alexa-488–conjugated anti-GPIb antibody. After laser injury, platelet accumulation and thrombus formation was assessed. (i) Representative images are shown, and data expressed as (ii) median thrombus area over time, and (iii) thrombus size determined by calculating the area under the curve. Between 8 and 10 thrombi were analyzed from 4 mice treated for each condition. **D**, Tail bleeding as determined as time to cessation of bleeding in mice pretreated with vehicle or 20 μmol/L 9-*cis*-RA for 10 min (n=10 for vehicle-treated and 9 for 9-*cis*-RA–treated samples). Unless stated otherwise, results are mean±SEM for n≥3. *P≤0.05 in comparison to vehicle controls.

As we have shown that RXR ligands affect several aspects of platelet activation, the effect of the 9-*cis*-RA on thrombus formation was assessed in vitro. Human whole blood was perfused over collagen-coated (100 μg/mL) Vena8 biochips for 10 minutes at an arterial shear rate of 20 dynes/cm^2^. Consistent with the observed inhibition of platelet activity, a significant reduction in thrombus formation (≈35%) was observed in 9-*cis*-RA–treated whole blood in comparison to vehicle-treated control samples (Figure 4A). To determine whether this inhibition of thrombus formation was because of a reduction in the ability of platelets to adhere to collagen, platelets were treated with integrilin, an antagonist of integrin α_IIb_β_3_, to prevent platelet–platelet interactions. As shown in Figure 4B, no significant difference in platelet adhesion on collagen was observed in platelets treated with 20 μmol/L 9-*cis*-RA compared with vehicle-treated controls, suggesting that 9-*cis*-RA does not affect adhesion to collagen under flow but instead inhibits thrombus growth. This was further supported by observations under static conditions, where treatment with 9-*cis*-RA resulted in an inhibition of the ability of platelets to spread on collagen-coated coverslips, but adhesion to collagen was not significantly altered (Figure IIIC).

### RXR Ligands Inhibit Hemostasis and Thrombosis in Mice

To determine the impact of RXR ligands on the acute regulation of platelet function in vivo, the effect of 9-*cis*-RA on laser-induced thrombosis in mouse cremaster muscle arterioles was explored. Figure 4C shows that although the initial kinetics of thrombus formation were similar, thrombi formed in 9-*cis*-RA–treated mice were consistently smaller (≈35%) than those formed after treatment with vehicle control (0.1% dimethyl sulfoxide; Figure 4Cii and 4Ciii). These results suggest an antithrombotic effect of RXR ligands in vivo. 9-*cis*-RA was also found to impair hemostasis in vivo as a significant increase in time to cessation of bleeding was seen after removal of the tail tip of mice treated with 9-*cis*-RA compared with vehicle control (Figure 4D).

### 9-*cis*-RA Does Not Modulate Early GPVI Signaling Events

We have shown that platelet activation after stimulation by GPVI agonists and thrombin is reduced after treatment with RXR ligands. To determine whether this was because of alterations in early signaling events, the phosphorylation levels of different signaling proteins involved in the GPVI and thrombin-signaling pathways were analyzed. Washed human platelets were pretreated with 9-*cis*-RA (10 and 20 μmol/L) or vehicle for 10 minutes before stimulation for 5 minutes by CRP-XL (1 μg/mL) or thrombin (0.1 U/mL). Higher concentrations of platelet agonists were used to enable phosphorylation of signaling components to be detected by Western blotting. Interestingly, despite the observed inhibition of platelet functions and thrombus formation, total tyrosine phosphorylation and phosphorylation of Syk and PLCγ2 (phospholipase C) after stimulation by CRP-XL were not altered by treatment with 9-*cis*-RA (10 and 20 μmol/L; Figure IVA and IVB in the online-only Data Supplement). PKC (protein kinase C) activity was also assessed using an antibody raised against the phosphorylated PKC substrate recognition sequence. PKC activity after stimulation by either CRP-XL or thrombin for 5 minutes was unaffected by treatment with 9-*cis*-RA (Figure IVC and IVD in the online-only Data Supplement) but was reduced at earlier time points, with inhibition observed at 90 seconds (CRP-XL) and 30 seconds (thrombin). These observations indicate that the RXR agonists cause a reduction in the rate but not the magnitude of PKC activation.

### 9-*cis*-RA Increases PKA Activity in Resting and Stimulated Platelets

The data presented here demonstrates a role for RXR in the negative regulation of platelet function, although this is not associated with prominent alterations in the early platelet signaling events associated with GPVI agonists or thrombin. Such broad effects are unlikely to be explained by inhibition of specific elements of activation pathways; therefore, activation of inhibitory pathways was investigated. Treatment of platelets with ligands for PPARs has previously been described to cause upregulation of PKA activity in some cases via increasing intracellular levels of cAMP.^2,4,12,27^ As we have identified that both PPARα and PPARγ can heterodimerize with RXR in platelets, we determined whether or not RXR agonists were capable of altering PKA activity.

**Figure 5.**
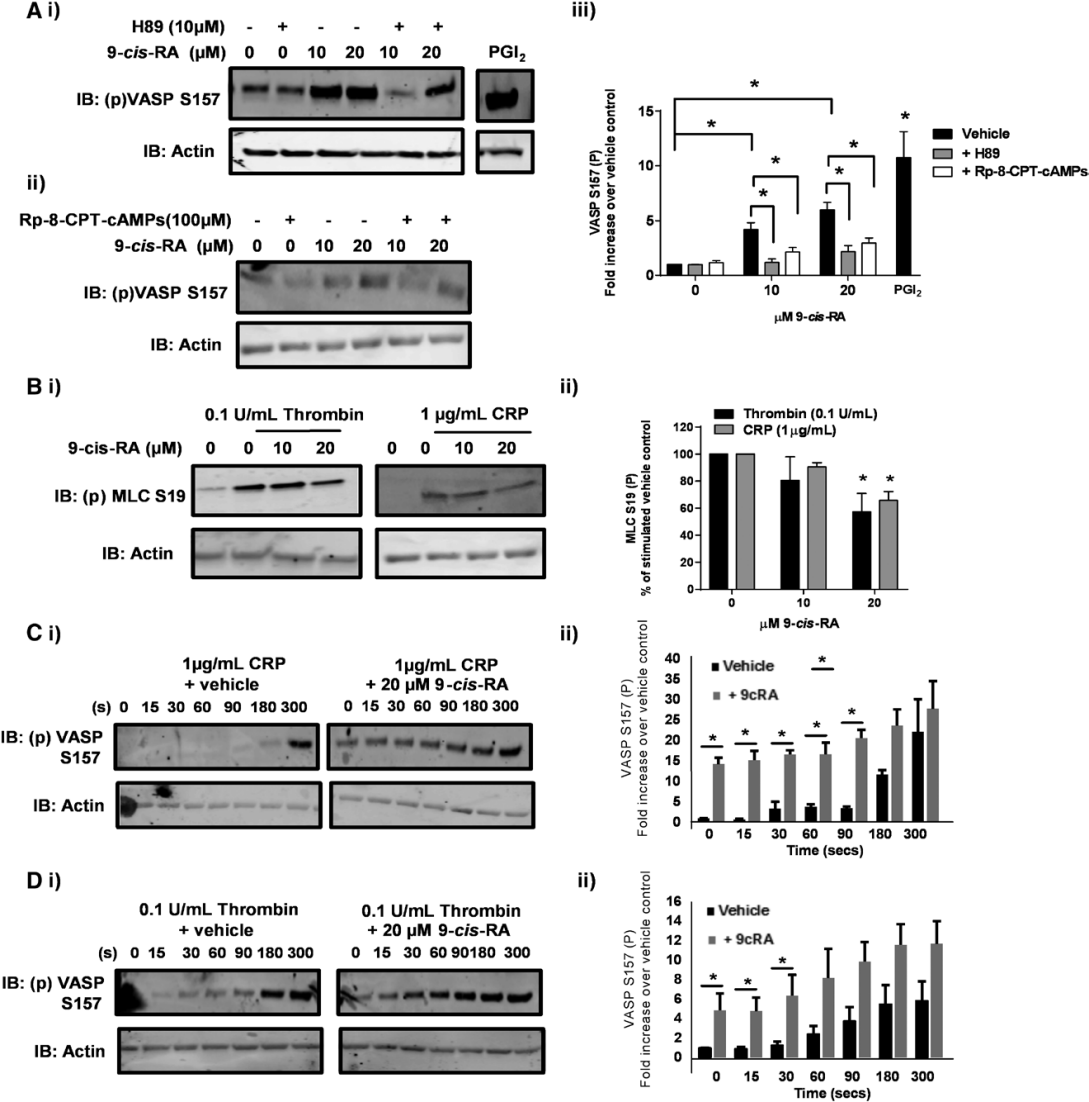
Retinoid X receptor (RXR) ligands and protein kinase A (PKA) activity. Resting human washed platelets were treated with 9-*cis*-retinoic acid (9-*cis*-RA; 10 and 20 μmol/L) in the presence or absence of (**A**) PKA inhibitors (i) H89 (10 μmol/L) or (ii) Rp-8-CPT-cAMPs (100 μmol/L) for 10 min and samples tested for VASP S157 phosphorylation, a marker of PKA activity. PGI_2_, which activates platelets through binding to the prostaglandin receptor (IP receptor) leading to activation of adenylyl cyclase, was included as a positive control for PKA activation. i and ii, Representative blots are shown and (iii) levels of phosphorylation were quantified and expressed as fold increase compared with vehicle control. **B**, Thrombin-stimulated (0.1 U/mL) or CRP-stimulated (1 μg/mL) platelets (0- and 180-s stimulation) pretreated with 9-*cis*-RA (10 and 20 μmol/L) were analyzed for myosin light chain (MLC) Ser19 phosphorylation. **C**, CRP-stimulated (1 μg/mL) or (**D**) thrombin-stimulated (0.1 U/mL) platelets (0, 15, 30, 60, 90, 180, and 300 s of stimulation) pretreated with 9-*cis*-RA (20 μmol/L) were also analyzed for VASP S157 phosphorylation. Blotting samples were lysed in Laemmli sample buffer before separation by SDS–PAGE and transferred onto polyvinylidene fluoride (PVDF) membranes. Actin was used as a loading control. i, Representative blots are shown and (ii) levels of phosphorylation were quantified and expressed as fold increase compared with vehicle control. Results are mean±SEM for n≥3. *P≤0.05 in comparison to vehicle controls.

To determine whether RXR agonists altered PKA activity, VASP (vasodilator-stimulated phosphoprotein) S157 phosphorylation (a PKA-specific phosphorylation site and marker of PKA activity) was measured. Unstimulated platelets treated with 9-*cis*-RA (10 and 20 μmol/L; Figure 5A) or methoprene acid (Figure V in the online-only Data Supplement; 10 and 20 μmol/L) showed increased phosphorylation of VASP S157 in comparison to untreated controls. 9-*cis*-RA–dependent increase in VASP S157 phosphorylation was prevented after treatment with 2 different PKA inhibitors H89 (10 μmol/L) and Rp-8-CPT-cAMPs (100 μmol/L; Figure 5A). Similar results were also observed after treatment with either docosahexaenoic acid or methoprene acid because both alternative RXR ligands also caused a significant increase in VASP S157 phosphorylation that was reversed after treatment with the PKA inhibitor H89 (Figure V in the online-only Data Supplement).

It has been shown that VASP can also be phosphorylated and regulated in platelets by the PKC isoforms and PKB/Akt. However, treatment with either a pan-PKC inhibitor GF109203X (10 μmol/L) or AKT inhibitor, AKT inhibitor IV (5 μmol/L), did not prevent the 9-*cis*-RA–induced increases in VASP S157 phosphorylation (Figure VI in the online-only Data Supplement), providing further support that RXR agonists exert their effect through PKA.

Activation of PKA has been shown to result in the negative regulation of the family of Rho GTPases including RhoA and Rac1 that have been identified as key regulators of platelet function. Negative regulation of RhoA, in turn, negatively regulates cytoskeleton rearrangements and phosphorylation of the myosin light chain. In further support of the inhibition of platelet function by 9-*cis*-RA being caused by an increase in PKA activity, we observed an inhibition of myosin light chain phosphorylation at Ser19 in both thrombin-treated (0.1 U/mL) and CRP-treated (1 μg/mL) platelets after treatment with 9-*cis*-RA (10 and 20 μmol/L) compared with vehicle controls (Figure 5B).

To investigate whether PKA activity was also altered in agonist-stimulated platelets, human washed platelets were pretreated with 9-*cis*-RA (20 μmol/L) before stimulation with CRP-XL (1 μg/mL) or thrombin (0.1 U/mL), and VASP S157 phosphorylation monitored over a period of 5 minutes. A time-dependent increase in VASP S157 phosphorylation was observed in both CRP-XL–stimulated and thrombin-stimulated platelets as has been described previously.^24^ As shown in Figure 5C and 5D VASP S157 phosphorylation was significantly increased in 9-*cis*-RA–treated platelets before stimulation with either CRP-XL or thrombin and remained significantly higher than vehicle control at early time points. At later time points, no significant difference in VASP S157 phosphorylation was observed in 9-*cis*-RA–treated platelets compared with controls. This suggests that RXR agonists may exert their inhibitory effects via elevation of PKA activity, whereas CRP and thrombin activate PKA as a form of negative feedback once platelet activation has occurred.

### RXR Ligand–Mediated Increases in PKA Activity Are Dependent on Adenylyl Cyclase Activity but Not Prostaglandin I2 Receptor Signaling

**Figure 6.**
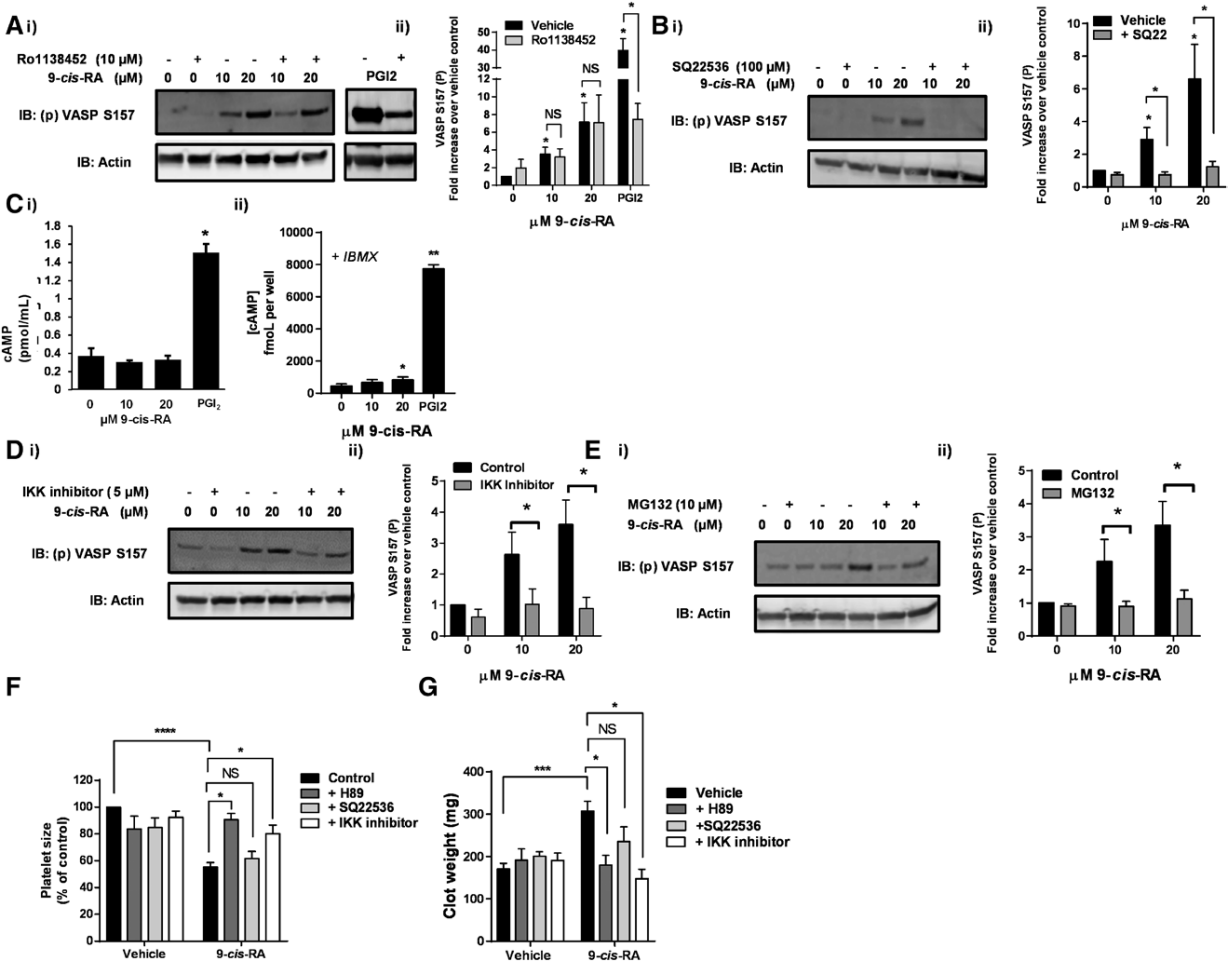
9-*cis*-retinoic acid (9-*cis*-RA)–dependent increase in protein kinase A (PKA) activity is dependent on cAMP and nuclear factor κ-light-chain-enhancer of activated B cell (NFκB). Resting human washed platelets were treated with 9-*cis*-RA (10 and 20 μmol/L) in the presence or absence of (**A**) IP receptor antagonist Ro1138452 (10 μmol/L), (**B**) adenylyl cyclase inhibitor SQ22536 (100 μmol/L), (**D**) IKK inhibitor (5 μmol/L), or (**E**) proteasome inhibitor MG132 (10 μmol/L) for 10 min, and samples tested for VASP S157 phosphorylation, a marker of PKA activity. Blotting samples were lysed in Laemmli sample buffer before separation on SDS–PAGE and transferred onto polyvinylidene fluoride (PVDF) membranes. Actin was used as a loading control. (i) Representative blots are shown and (ii) levels of phosphorylation were quantified and expressed as fold increase compared with vehicle control. **C**, Levels of cAMP were determined in 9-*cis*-RA–treated (10 and 20 μmol/L) resting washed platelets using a cAMP ELISA assay kit (as per manufacturer’s instructions) in the (i) absence and (ii) presence of IBMX (1 mmol/L). PGI2 was included as a positive control for PKA activation and intracellular increases in cAMP. **F** and **G**, Human washed platelets were treated with 9-*cis*-RA (20 μmol/L) in the presence or absence of H89 (10 μmol/L), IKK inhibitor (5 μmol/L) or SQ22536 (100 μmol/L) for 10 min (**F**) before exposure to fibrinogen-coated (100 μg/mL) coverslips and left to spread and adhered for 45 min. Data presented as average platelet size as calculated by looking at individual platelet surface area of platelets adhered in 5 randomly selected fields of view using ImageJ software. The larger the surface area, the more spread the platelet is. **G**, In the presence of 2 mg/mL fibrinogen and 2 mmol/L CaCl_2_, in aggregometer tubes, clot retraction was initiated by addition of thrombin 1 U/mL (final concentration) and left to proceed for 1 h at room temperature. Extent of clot retraction was determined by comparing clot weight. Results are mean±SEM for n≥3. *P≤0.05 in comparison to vehicle controls.

Traditionally, activation of PKA occurs after the production of cAMP downstream of prostaglandin I2 (IP) receptor signaling. Binding of PGI_2_ to the IP receptor causes Gs-coupled signaling that activates adenylyl cyclase and stimulates the production of cAMP, which then activates PKA. RXR ligand–mediated increases in PKA activity were found to be independent of IP receptor activation, as the IP receptor antagonist Ro1138452 (10 μmol/L)^28,29^ was unable to reverse 9-*cis*-RA–mediated increases in VASP S157 phosphorylation (Figure 6A). However, RXR ligand–dependent activation of PKA was attenuated after treatment with SQ22536 (100 μmol/L), an adenylyl cyclase inhibitor, because 9-*cis*-RA–mediated (and docosahexaenoic acid–mediated) increases in VASP S157 phosphorylation were reduced after pretreatment with SQ22536 (Figure 6B). Interestingly, however, under the same experimental conditions, treatment with 9-*cis*-RA did not alter cAMP levels in resting platelets (Figure 6Ci). In the presence of the phosphodiesterase inhibitor, IBMX (1 mmol/L), a minor increase in cAMP was observed after treatment with 9-*cis*-RA (Figure 6Cii) but not after treatment with methoprene acid or docosahexaenoic acid (Figure VIIA and VIIB in the online-only Data Supplement). These data suggest that RXR ligand–dependent increases in VASP S157 phosphorylation and PKA activity are dependent on intracellular cAMP but not associated with significant increases in cAMP levels.

### 9-*cis*-RA–Induced Increases in PKA Activity Are Associated With NFκB Activity

In addition to cAMP-mediated activation of PKA, PKA has also been shown to be activated via a mechanism that is dependent on NFκB, after stimulation of platelets by thrombin or collagen.^24^ In this mechanism, a subpopulation of PKA is associated with NFκB–IκBα and after stimulation by collagen or thrombin, NFκB is activated, leading to degradation and release of IκBα.^30^ This enables dissociation of the catalytic subunit of PKA from NFκB–IκBα, resulting in the activation of PKA. To investigate whether RXR agonists activate PKA via NFκB–IκBα, we treated platelets with IKK inhibitor VII (5 μmol/) to determine whether it was able to reverse the observed platelet inhibition. IKK inhibitor VII was used previously to establish the role of NFκB in PKA activation and prevents the degradation and release of IκBα. IKK inhibitor VII prevented 9-*cis*-RA–induced VASP S157 phosphorylation in resting platelets, highlighting a role for the activation of NFκB and dissociation of IκBα in RXR-ligand–induced regulation of PKA activity (Figure 6D). Similar observations were also made after treatment with docosahexaenoic acid and methoprene acid (Figure VIIC and VIID in the online-only Data Supplement). Furthermore, treatment with a proteasome inhibitor, MG132 (10 μmol/L), used to prevent degradation of IκBα, also prevented 9-*cis*-RA–induced phosphorylation of VASP S157 (Figure 6E). Both H89 and the IKK inhibitor, but not SQ22536 the adenylyl cyclase inhibitor, were found to reverse 9-*cis*-RA–mediated inhibition of platelet spreading on fibrinogen and clot retraction, implicating RXR-dependent upregulation of NFκB–IκBα and PKA in the negative regulation of platelet αIIbβ_3_ outside-in signaling (Figure 6F and 6G).

We have previously described that PPARγ ligands are capable of negatively regulating integrin αIIbβ3 outside-in signaling through upregulation of PKA activity.^12^ As we found PPARγ can be coimmunoprecipitated with RXR from platelets, we determined whether it is the activation and modulation of RXR:PPARγ heterodimers that regulates the NFκB-mediated upregulation of PKA activity. However, the IKK inhibitor was unable to reverse 15dPGJ2-mediated (an endogenous ligand for PPARγ) increases in VASP S157 phosphorylation (Figure VIIIA in the online-only Data Supplement), suggesting that PPARγ-dependent increases in PKA activity are not linked to the regulation of NFκB. In further support of this, the IKK inhibitor was also unable to reverse the increase in VASP S157 phosphorylation and PKA activity observed after treatment with the RXR modulator LG101506, which specifically modulates RXR:PPAR heterodimers (Figure VIIIB in the online-only Data Supplement). This suggests that ligands of RXR and PPARγ upregulate PKA activity via different mechanisms.

## Discussion

RXRs are intracellular nuclear receptors expressed in human platelets where they function to negatively regulate platelet responses to agonists.^3,4,11,21^ In its genomic role, RXR is thought to heterodimerize with several intracellular nuclear receptors including PPARs, LXR, and FXR and is, therefore, also associated with the regulation of glucose, triglyceride, cholesterol, and bile acid homeostasis.^20^ Dysregulation of these homeostatic control pathways can result in several metabolic disorders including obesity, type 2 diabetes mellitus, hyperlipidemia, atherosclerosis, and cardiovascular disease. Treatment with RXR ligands shows some efficacy at reducing the progression of atherosclerosis in apolipoprotein E knockout mice.^18^ Alitretinoin, the commercial name for RXR ligand 9-*cis*-RA, is used for the treatment of Kaposi sarcoma and eczema, and decreased blood clotting is currently listed as one of its side effects.^18,22,23^ In support of the ability of 9-*cis*-RA to reduce blood clotting, we show that treatment with 9-*cis*-RA increases bleeding time in mice and elicits other antithrombotic effects, which we attribute to an activation of PKA via a mechanism that requires cAMP and involves NFκB. As platelets play an important role in the pathogenesis of cardiovascular disease, our findings that RXR ligands inhibit platelet activation and thrombus formation may help to explain the efficacy of these compounds in vivo.

We observed that treatment of platelets with RXR agonists 9-*cis*-RA, methoprene acid, and docosahexaenoic acid caused a significant inhibition of α-granule secretion; mobilization of intracellular calcium; platelet aggregation and integrin α_IIb_β_3_ activation to several platelet agonists including collagen, CRP-XL, and thrombin; and an inhibition of integrin α_IIb_β_3_ outside-in signaling. This inhibition of platelet activity by 9-*cis*-RA also correlated with inhibition of thrombus formation in vitro and in vivo, suggesting that the previously described cardioprotective effects of RXR agonists in reducing atherosclerosis^18^ could also potentially be attributed to their negative regulation of platelet function.

Treatment of platelets with ligands for PPARs, PPARα and PPARβ/δ or PPARγ common binding partners for RXR (Figure 1) have been previously described to cause upregulation of cAMP levels or activation of PKA. RXR ligand–stimulated inhibition of platelet function and thrombus formation were also found to be associated with an upregulation of PKA activity, as treatment of platelets with RXR agonists resulted in an increase in VASP phosphorylation at S157 (the PKA phosphorylation site) in both resting and agonist-stimulated platelets, and this increase was reversed after treatment with the PKA inhibitors H89 and Rp-8-CPT-cAMPs or adenylyl cyclase inhibitor SQ22358 but not after treatment with an IP receptor antagonist (Ro1138452). This suggests that RXR agonists activate PKA through a mechanism that is dependent on cAMP, although no major alterations in cAMP levels were observed after treatment with the different RXR agonists. It is possible that treatment with RXR agonists does cause small increases in platelet cAMP levels that are not detected given current limitations in sensitivity of the assays used. It has been shown that even minor increases in cAMP levels can cause significant activation of cellular PKA and large increases in VASP S157 phosphorylation.^31^ As such, a role for RXR ligands in the upregulation of adenylyl cyclase activity cannot be ruled out. It is, however, interesting to note that RXR ligand–mediated inhibition of integrin αIIbβ3 outside-in signaling cannot be reversed by inhibition of adenylyl cyclase, suggesting a role for another PKA-linked signaling pathway in this negative regulation of platelet function.

It has been previously shown that activation of NFκB is involved in regulation of PKA activity after platelet activation by collagen or thrombin, where the inactive NFκB–IκBα complex binds to and inactivates the PKA catalytic subunit.^24^ In cancer cells, activation of NFκB has also been shown to occur after treatment with RXR ligands, including 9*-cis*-RA, and inhibition or downregulation of NFκB has been shown to reduce RXR-mediated cell differentiation and apoptosis of cancerous cells.^32^ We, therefore, hypothesized that treatment with RXR ligands could enable targeted degradation of the inactive NFκB–IκBα complex, releasing the PKA catalytic subunit and relieving the inhibition of PKA, resulting in an increase in PKA activity and subsequent substrate phosphorylation. This was supported by observations that treatment with H89 and the IKK inhibitor prevented 9-*cis*-RA–mediated upregulation of PKA activity and also reversed 9-*cis*-RA–mediated inhibition of platelet spreading on fibrinogen and clot retraction, which is dependent on integrin αIIbβ3 outside-in signaling. It is interesting to note, however, that the upregulation of PKA activity via NFκB is not a mechanism that is shared with the PPARs, typical binding partners of RXR, as the increase in VASP S157 phosphorylation observed after treatment with the RXR:PPAR modulator LG101506 could not be reversed by pretreatment with the IKK inhibitor.

Activation of PKA has been shown to negatively regulate the family of Rho GTPases including RhoA and Rac1.^24,33,34^ Rho GTPases including Rac1 are key mediators of platelet function, and mice deficient in Rac1 display major defects in platelet activation downstream of both GPVI and GPCRs and reduced thrombus formation in vivo.^35–37^ Previously published work suggests that Rac1 activity is reduced after treatment of platelets with RXR ligands, and this is attributed to an interaction of RXR with Gq.^5^ In agreement with alteration of RhoA activity, a reduction in myosin light chain phosphorylation was observed after treatment with 9-*cis*-RA. The upregulation of PKA activity that we describe here could also provide an additional mechanism that underlies this reduction in Rac1 activity that may explain the broad-spectrum inhibition by RXR ligands of platelet activity to multiple platelet agonists. Because RXR has the potential to form heterodimers with several different nuclear receptors, multiple mechanisms of regulation could exist.

The data presented here significantly build on previously observed inhibitory effects of RXR ligands on platelet activity^5^ and highlight a relatively unknown mechanism of PKA activation as a potential target for antiplatelet therapy. The data presented here suggest that RXR ligands could offer extra protective effects in vivo if developed as drug targets for the treatment of other diseases such as diabetes mellitus, although these effects would need to be carefully balanced to ensure there is no increased risk of bleeding.

## Acknowledgments

A.J. Unsworth designed the research, performed experiments, analyzed results, and wrote the article. G.D. Flora designed the research, performed experiments, and analyzed results. A.P. Bye performed experiments, analyzed results, and wrote the article, P. Sasikumar, N. Kriek, T. Sage, and M. Crescente performed experiments. J.M. Gibbins designed the research and wrote the article.

## Sources of Funding

This work was supported by the British Heart Foundation (RG/09/011/28094, RG/15/2/31224, and PG/15/21/31355), a Felix Scholarship, and the Medical Research Council (MR/J002666/1).

## Disclosures

None.

## Supplementary Material

## Highlights

- Platelets express the retinoid X receptors (RXRα and RXRβ).
- RXR ligands cause a widespread inhibition of platelet function to multiple platelet agonists.
- RXR ligands inhibit thrombus formation and impair hemostasis.
- This nongenomic regulation by RXR ligands is mediated by activation of PKA that involves cAMP and nuclear factor κ-light-chain-enhancer of activated B cell.
