# Supplementary material for: RXR Ligands Negatively Regulate Thrombosis and Hemostasis
Source: Arterioscler Thromb Vasc Biol. 2017 Mar 2;37(5):812–22. doi: 10.1161/ATVBAHA.117.309207 (PMC5405776; doi:10.1161/ATVBAHA.117.309207)
Supplement: Supplementary file 1 [file atv-37-812-s001.pdf]

MATERIALS AND METHODS

RXR ligands negatively regulate thrombosis and haemostasis.

A.J. Unsworth, G. Flora, P. Sasikumar, A.P. Bye, T. Sage, N. Kriek, M.Crescente, J.M. Gibbins.

MATERIALS AND METHODS

Reagents

9-*cis*-retinoic acid, docosahexaenoic acid, methoprene acid, bovine thrombin, H89, SQ22536 and MG132 were purchased from Sigma Aldrich (Poole, UK). Rp-8-CPTs-cAMP and Ro1138452 were purchased from Tocris. Horm collagen was purchased from Nycomed, Austria, CRP-XL from Prof. R Farndale (University of Cambridge, UK). Primary anti- RXR, Syk (N-19), PLCγ2 (Q20) and actin (C11) antibodies were purchased from Santa Cruz Biotechnology (Calne, UK). Anti-Phospho–PKC substrate antibody, phospho-myosin light chain S19 and phospho-Ser157 VASP antibodies were purchased from New England BioLabs, USA (Cell Signalling Hitchin, UK), anti-phospho-Tyr 4G10 antibody and IKK inhibitor VII were purchased from Millipore (Watford, UK). Fluorophore conjugated secondary antibodies, Fura-2AM calcium indicator dye and Alexa-488 conjugated phalloidin were purchased from Life Technologies (Paisely, UK). All other reagents were from previously described sources [1, 2].

Platelet preparation

Human blood was obtained from consenting aspirin-free, healthy volunteers following procedures approved by the University of Reading Research Ethics Committee. Blood was collected into 3.8% (w/v) sodium citrate before mixing with acid citrate dextrose (29.9 mM $Na_3C_6H_5O_7$, 113.8 mM glucose, 72.6 mM NaCl, and 2.9 mM citric acid [pH 6.4]). Human washed platelets were prepared by centrifugation as described previously [3]. Platelets were resuspended in modified Tyrode's-HEPES buffer, (134mM NaCl, 0.34mM Na_2HPO_4, 2.9mM KCl, 12mM $NaHCO_3$, 20mM N-2-hydroxyethylpiperazine-N-2-ethanesulfonic acid, 5mM glucose and 1mM $MgCl_2$, pH 7.3) and rested for 30 minutes at 30°C before use.

Immunofluorescence microscopy

Human and mouse platelets stimulated with or without U46619 (3µM) were left to settle on poly-L-lysine coverslips for 1 hour at 37ºC before permeabilisation and blocking (0.2% Triton-X-100, 1% BSA, 2% donkey serum). Coverslips were then incubated with primary antibodies for RXR and CD41 (a marker of the platelet membrane) overnight at 4ºC and washed in PBS before staining with Alexa-fluorophore conjugated secondary antibodies (488 nm and 647 nm) for 1 hour at room temperature in the dark. Coverslips were washed and mounted onto slides. Platelets were imaged with a 100 x magnification oil immersion lens on a Nikon A1-R confocal microscope.

Platelet aggregation

Aggregation of human washed platelets was measured by optical aggregometry (Chrono-log Corp., Havertown, PA, USA) as described previously [4].

Fibrinogen binding and alpha granule secretion

Activation of the integrin $\alpha_{IIb}\beta_3$ and alpha granule secretion were measured by detecting levels of fibrinogen binding and P-selectin exposure at the platelet surface by flow cytometry using fluorescein isothiocyanate-labelled (FITC) anti-fibrinogen antibody and PE/Cy5 anti-human CD62P respectively. Using a BD Accuri C6 flow cytometer, 5,000 events were analysed using the CFlow Sampler software as described previously [5].

Intracellular Calcium Levels

PRP was loaded with Fura-2 AM (2 µM) for 1h at 30ºC and then washed by centrifugation at 350 *xg* for 20 mins and resuspended in Tyrode's-HEPEs buffer containing 0.4 U/ml apyrase. Fura-2AM loaded platelets were incubated with inhibitors or vehicle at 37ºC for 10 minutes prior to addition of agonists. Fluorescence measurements with excitation at 340 and 380 nm

and emission at 510 nm were recorded over a period of 5 mins using a NOVOstar plate reader (BMG Labtech). ($[Ca^{2+}]_i$ was estimated using the ratio of the 340 and 380 nm excited signals, using the method of Grynkiewicz *et al* [6] and $[Ca^{2+}]_i$ concentrations were calculated as described previously [7, 8].

Adhesion and spreading on fibrinogen

Washed platelets (2 x 10^7 cells/mL), treated with or without RXR ligands were exposed to fibrinogen (100 µg/ml) coated coverslips and incubated for 45 minutes at 37°C. Non adherent platelets were removed before fixing using 0.2% paraformaldehyde solution. Adhered platelets were permeabilised with 0.1% Triton-X-100 prior to staining with Alexa 488 conjugated-phalloidin for 1 hr at room temperature. Adherent platelets were then imaged with a 100 x magnification oil immersion lens on a Nikon A1-R confocal microscope. Adhesion and spreading data in each experiment were obtained by counting, for each sample, the number of platelets in 5 randomly chosen fields of view. The number of platelets generating filopodia or lamellipodia were also counted, and platelets scored as being adhered but not spread, extending filopodia and fully spread (formation of lamellipodia) and the relative frequency determined.

Clot retraction assay

Human washed platelets at 5 × 10^8/mL were added to aggregometer tubes in the presence of 2 mg/mL fibrinogen and 2 mM $CaCl_2$. Clot formation was initiated by adding an equal volume of 2 U/mL thrombin and allowed to progress for 1 hour at room temperature. Weight of the clot and volume of extruded serum were measured.

Thrombus formation on collagen

Thrombus formation in the presence or absence of integrillin (10 µM) was studied *in vitro* using microfluidic flow cells (Vena8, CellixLtd, Dublin, Ireland) coated with collagen (100 µg/mL). Blood was passed through the flow cells at an arterial shear rate of 20 dyn/cm^2 as described previously [5].

Tail bleeding assay

Tail bleeding experiments were performed on 20–35 g male mice, anesthetized with ketamine (100 mg/kg) and xylazine (10 mg/kg) injected intraperitoneally. RXR ligand 9-*cis*-RA (20 µM) or vehicle control (DMSO 0.1% v/v), calculated by taking into consideration mouse weight and blood volume, was injected into the femoral vein 10 minutes prior to removal of the tip of the tail using a sharp razor blade. The tail tip was then placed in sterile saline (37 ºC) and time to cessation of bleeding (secs) measured.

Laser injury induced thrombus formation

In vivo thrombosis was assayed using a laser injury model by intravital microscopy as described previously [9]. In brief, treatment with RXR ligand 9-*cis*-RA (20 µM) or vehicle control (DMSO 0.1% v/v), as calculated by taking into consideration mouse weight and blood volume, was administered intravenously to mice and platelets fluorescently labelled by injection of Alexa 488-conjugated anti GPIb antibody for 10 minutes prior to laser injury. After laser induced injury of the inner wall of the cremaster muscle arterioles, accumulation of platelets was assessed. Fluorescence and brightfield images were recorded using an Olympus BX61W microscope with a 60 x/1.0 NA water immersion objective and a high speed camera, and data analyzed using Image J software.

Immunoblotting and immunoprecipitation

Washed platelets (4 × 10^8 cells/mL) were lysed in an equal volume of NP40 buffer (300 mM NaCl, 20 mM Tris base, 2 mM EGTA, 2 mM EDTA, 1 mM PMSF, 10 µg/ml aprotinin, 10

µg/ml leupeptin, 0.7µg/ml pepstatin A, 2mM sodium orthovanadate, 2% NP-40, pH 7.3), and proteins of interest were isolated using 1 µg/mL of appropriate antibodies as described previously [4]. Immunoblotting was performed using standard techniques as described previously [3].
Levels of phosphorylated proteins were detected using fluorophore conjugated secondary antibodies and visualised using a Typhoon Trio Fluorimager and Image Quant software (GE Healthcare). Band intensities were quantified and levels of the immunoprecipitated protein were used to control for protein loading using Image Quant software.

Detection of cAMP levels.

Human washed platelets (2×10^8 cells/mL) were treated with RXR ligands in the presence and absence of phosphodiesterase inhibitor IBMX (1mM) for 10 minutes, lysed in lysis buffer provided and cAMP levels measured using a cAMP ELISA kit (ENZO Life sciences) and (GE Healthcare) respectively as per manufacturers instructions, and as described previously [10] [11].

Statistical analysis

Statistical analyses were performed using GraphPad prism software. Data were analysed using student T-test and if more than two means were present, significance was determined by one way ANOVA. Values obtained in several experiments were converted into percentages for comparison of controls with treated samples or expressed as fold change compared to control. Where data was normalised, statistical analysis was performed prior to normalisation and also using the non-parametric Wilcoxon signed-rank test. $P \leq 0.05$ was considered statistically significant. Unless stated otherwise, values are expressed as mean ±SEM, n values are ≥ 3.

1. Bye AP, Unsworth AJ, Vaiyapuri S, et al., Ibrutinib Inhibits Platelet Integrin alphaIIbbeta3 Outside-In Signaling and Thrombus Stability But Not Adhesion to Collagen. Arterioscler Thromb Vasc Biol, 2015.
2. Jones CI, Moraes LA, and Gibbins JM, Regulation of platelet biology by platelet endothelial cell adhesion molecule-1. Platelets, 2012; 23(5):331-5.
3. Kaiser WJ, Holbrook LM, Tucker KL, Stanley RG, and Gibbins JM, A functional proteomic method for the enrichment of peripheral membrane proteins reveals the collagen binding protein Hsp47 is exposed on the surface of activated human platelets. J Proteome Res, 2009; 8(6):2903-14.
4. Moraes LA, Spyridon M, Kaiser WJ, et al., Non-genomic effects of PPARgamma ligands: inhibition of GPVI-stimulated platelet activation. J Thromb Haemost, 2010; 8(3):577-87.
5. Vaiyapuri S, Jones CI, Sasikumar P, et al., Gap junctions and connexin hemichannels underpin hemostasis and thrombosis. Circulation, 2012; 125(20):2479-91.
6. Grynkiewicz G, Poenie M, and Tsien RY, A new generation of Ca2+ indicators with greatly improved fluorescence properties. J Biol Chem, 1985; 260(6):3440-50.
7. Poenie M, Alteration of intracellular Fura-2 fluorescence by viscosity: a simple correction. Cell Calcium, 1990; 11(2-3):85-91.
8. Bye AP, Unsworth AJ, Vaiyapuri S, et al., Ibrutinib Inhibits Platelet Integrin alphaIIbbeta3 Outside-In Signaling and Thrombus Stability But Not Adhesion to Collagen. Arterioscler Thromb Vasc Biol, 2015; 35(11):2326-35.
9. Spyridon M, Moraes LA, Jones CI, et al., LXR as a novel antithrombotic target. Blood, 2011; 117(21):5751-61.

10. Moraes LA, Unsworth AJ, Vaiyapuri S, et al., Farnesoid X Receptor and Its Ligands Inhibit the Function of Platelets. Arterioscler Thromb Vasc Biol, 2016.
11. Aburima A, Wraith KS, Raslan Z, et al., cAMP signaling regulates platelet myosin light chain (MLC) phosphorylation and shape change through targeting the RhoA-Rho kinase-MLC phosphatase signaling pathway. Blood, 2013; 122(20):3533-45.
