# Supplementary material for: RXR Ligands Negatively Regulate Thrombosis and Hemostasis
Source: Arterioscler Thromb Vasc Biol. 2017 Mar 2;37(5):812–22. doi: 10.1161/ATVBAHA.117.309207 (PMC5405776; doi:10.1161/ATVBAHA.117.309207)

SUPPLEMENTAL MATERIAL

RXR ligands negatively regulate thrombosis and haemostasis.

A.J. Unsworth, G. Flora, P. Sasikumar, A.P. Bye, T. Sage, N. Kriek, M.Crescente, J.M. Gibbins.

SUPPLEMENTAL MATERIAL

Supplemental Figure I. Washed human platelets were A) treated with i) 9-*cis*-RA, ii) synthetic RXR agonist methoprene and iii) endogenous RXR agonist docosahexaenoic acid and their ability to stimulate platelet aggregation in the absence of platelet agonist monitored using optical light transmission aggregometry, representative traces shown. Human washed platelets were B,C) pre-treated with methoprene acid (10, 20 μM) or D,E) pre-treated with docosahexaenoic acid (10, 20 μM) for 10 minutes prior to stimulation with either B,D) collagen (1 μg/mL) or C,D) thrombin (0.05 U/mL) and aggregation monitored using optical light transmission aggregometry i) Representative traces and ii) quantified data shown. F) pre-treated with 9-*cis*-RA (20 μM) for 10 minutes or vehicle control, in the presence of P”y12 inhibitor, cangrelor (1 μM), P2Y1 inhibitor, MRS2179 (100 μM) and cyclooxygenase inhibitor, indomethacin (20 μM) quantified data shown. G) pre-treated with RXR antagonist HX531 (10, 30 μM) for 10 minutes and aggregation to i) collagen (0-10 μg/mL), ii) thrombin (0-1 U/mL), iii) U46619 (0-3 μM) was monitored using an optical light transmission plate based aggregometry assay, quantified data shown. Data expressed as a percentage of vehicle treated control, results are mean + S.E.M. for n≥3, * indicates p≤0.05 in comparison to vehicle controls.

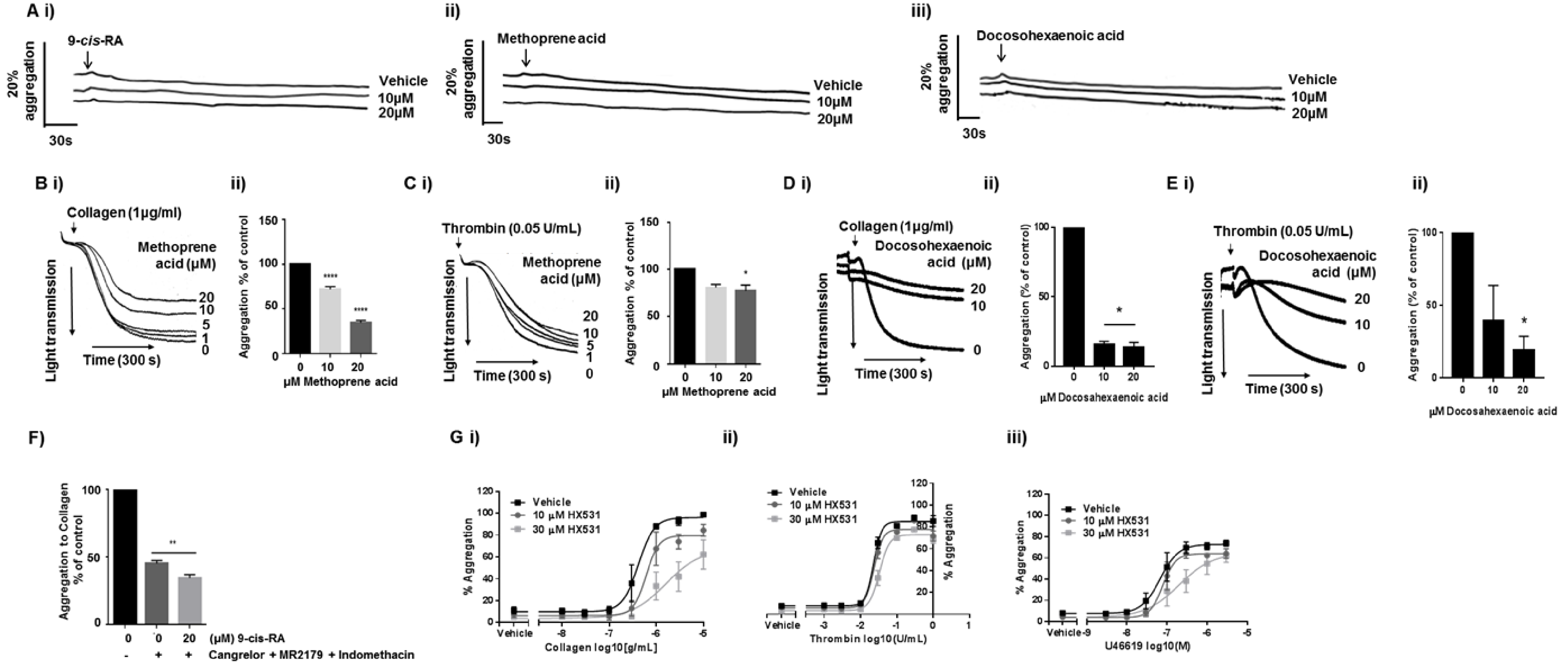

Supplemental Figure II. The effect of methoprene acid on platelet function. Human washed platelets were pre-treated for 10 minutes with increasing concentrations of methoprene acid (10, 20 µM) before stimulation by CRP-XL (0.25 µg/mL) or thrombin (0.05 U/mL). A) Platelet activation measured as fibrinogen binding in i) CRP-XL stimulated and ii) thrombin stimulated platelets. B) Alpha granule secretion as P-selectin exposure in i) CRP-XL and ii) thrombin stimulated platelets. Mobilisation of intracellular calcium determined in Fura-2 AM loaded platelets following stimulation with C) CRP-XL, D) thrombin. i) Representative traces and ii) quantified data shown. Data expressed as a percentage of untreated control. Results are mean + S.E.M. for n≥3, * indicates p≤0.05 in comparison to vehicle controls.

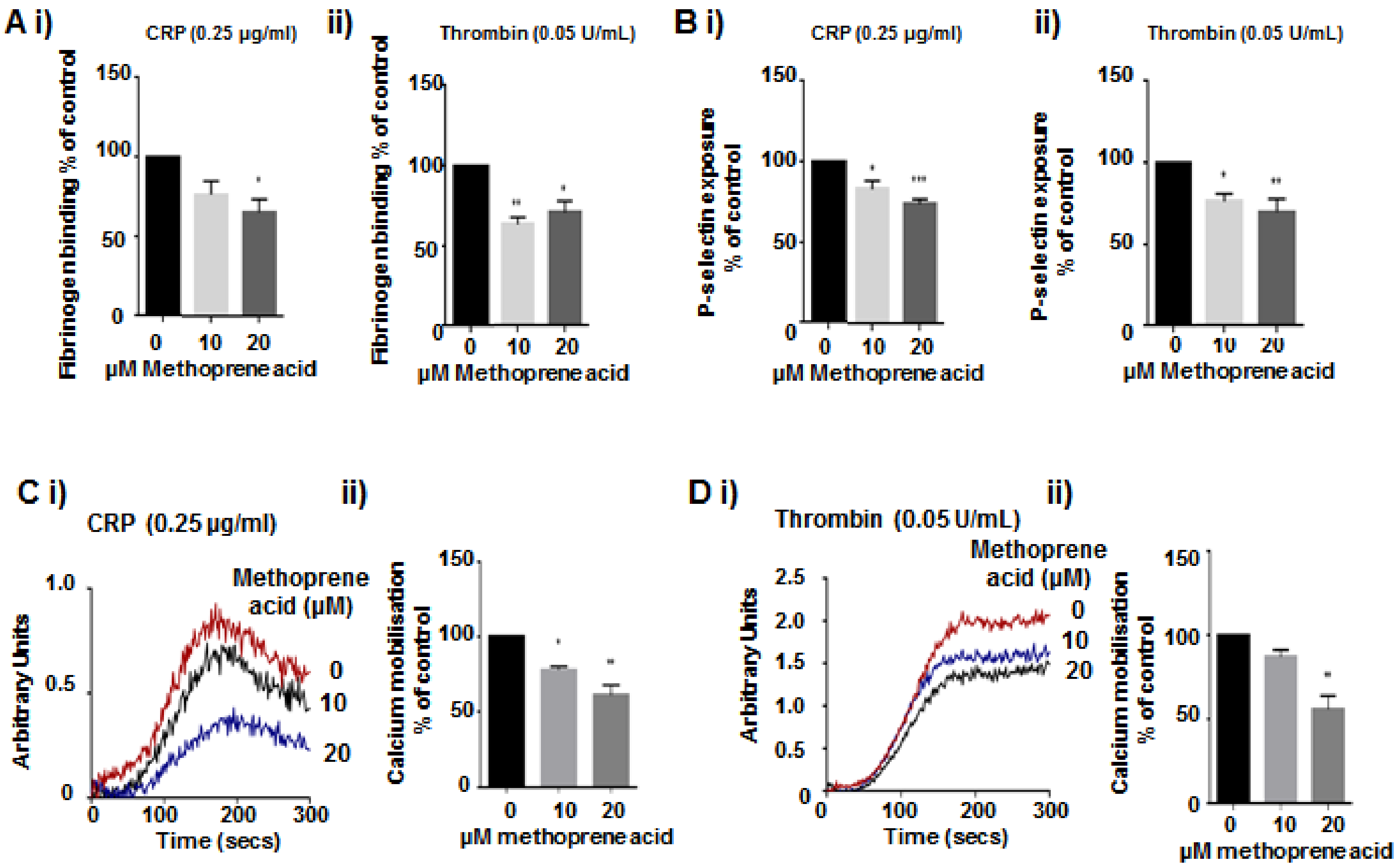

Supplemental Figure III. The effect of RXR ligands on adhesion and spreading Human washed platelets pre-treated for 10 minutes with docosahexaenoic acid (20 µM) or vehicle control were exposed to fibrinogen (100 µg/mL) coated coverslips. B) Clot retraction, human washed platelets pre-treated for 10 minutes with increasing concentrations of i) docosahexaenoic acid (10, 20 µM), ii) methoprene acid (10, 20 µ or vehicle control were added to aggregometer tubes in the presence of 2 mg/mL fibrinogen and 2 mM $CaCl_2$. Clot retraction was initiated by addition of thrombin 1 U/mL (final concentration) and left to proceed for 1 hour at room temperature. Extent of clot retraction was determined by comparing clot weight. i) Representative images using red blood cell stained PRP, ii) data expressed as clot weight (mg). C) Human washed platelets pre-treated for 10 minutes with 9-*cis*-RA (20 µM) or vehicle control were exposed to collagen (100 µg/mL) coated coverslips. A,C i) Representative images of spreading and adhesion after 45 min. Platelets were stained with phalloidin Alexa-488 for visualisation. Images were taken under oil immersion lens with magnification ×100. ii) Adhesion, number of platelets adhered were counted in 5 randomly selected fields of view and the number of cells adhered expressed as a percentage of the vehicle treated control. iii) Spreading, platelets were classified into 3 different categories to determine the extent of their spreading (Adhered but not spread, Filopodia: platelets in the process of extending filopodia and Lamellipodia: platelets in the process of extending lamellipodia including those fully spread). Results expressed, as a percentage of the total number of platelets adhered. Unless stated otherwise results are mean + S.E.M. for n≥3, * indicates p≤0.05 in comparison to vehicle controls.

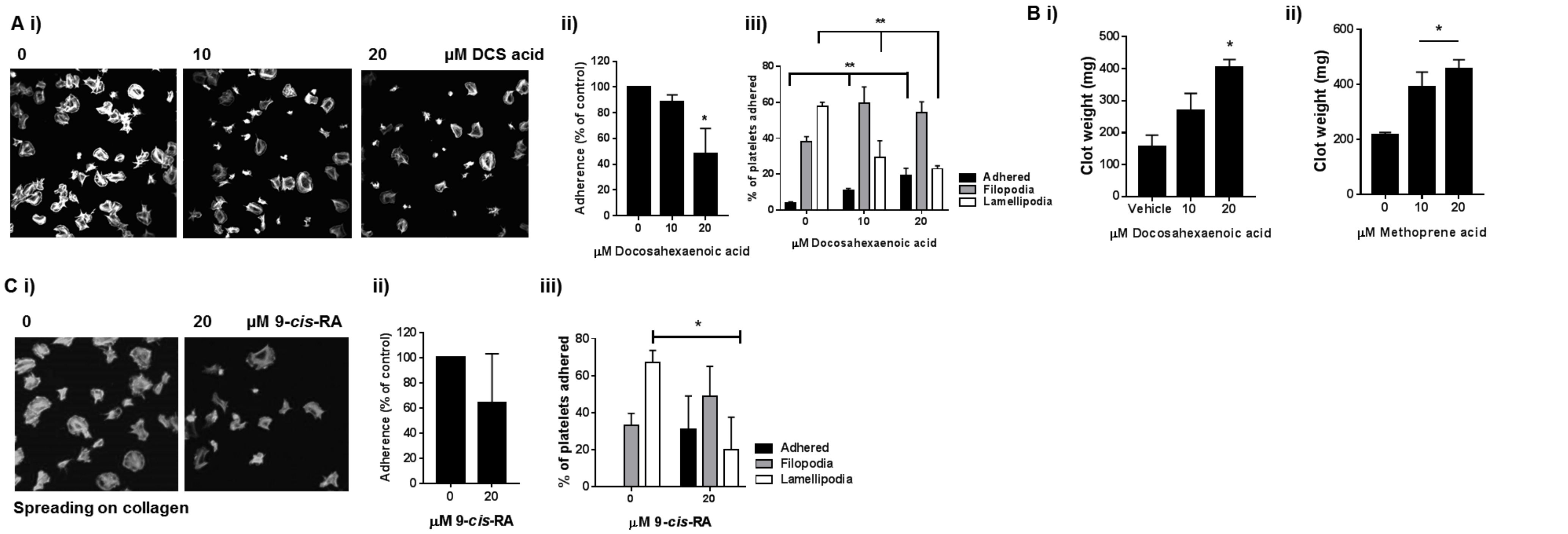

Supplemental Figure IV. Signalling in 9-*cis*-RA treated platelets. 9-*cis*-RA, (0, 10, 20 µM) pre-treated human washed platelet lysates were tested for A) global tyrosine phosphorylation and B) Syk and PLCγ2 phosphorylation in CRP-XL (1 µg/mL) stimulated platelets. PKC substrate phosphorylation C) after 90 and 300 secs of CRP-XL (1 µg/mL) stimulation and D) 30 and 300 secs of thrombin stimulation (0.1 U/mL). Samples were lysed in Laemmli sample buffer, separated by SDS PAGE and transferred to PVDF membranes before blotting with 4G10 antibody to measure global tyrosine phosphorylation or a phospho-site specific antibody against the PKC substrate recognition sequence. Syk and PLCγ2 were immunoprecipitated from lysates prior to addition of Laemmli sample buffer. Blots were reprobed for total Syk, PLCγ2 or actin to confirm equal loading. A-D) representative blots shown, C-D) i) representative blots shown, ii) Levels of total phosphorylation were quantified and expressed as a percentage of vehicle treated controls. Results are mean + S.E.M. for n≥3, * indicates p≤0.05 in comparison to vehicle controls.

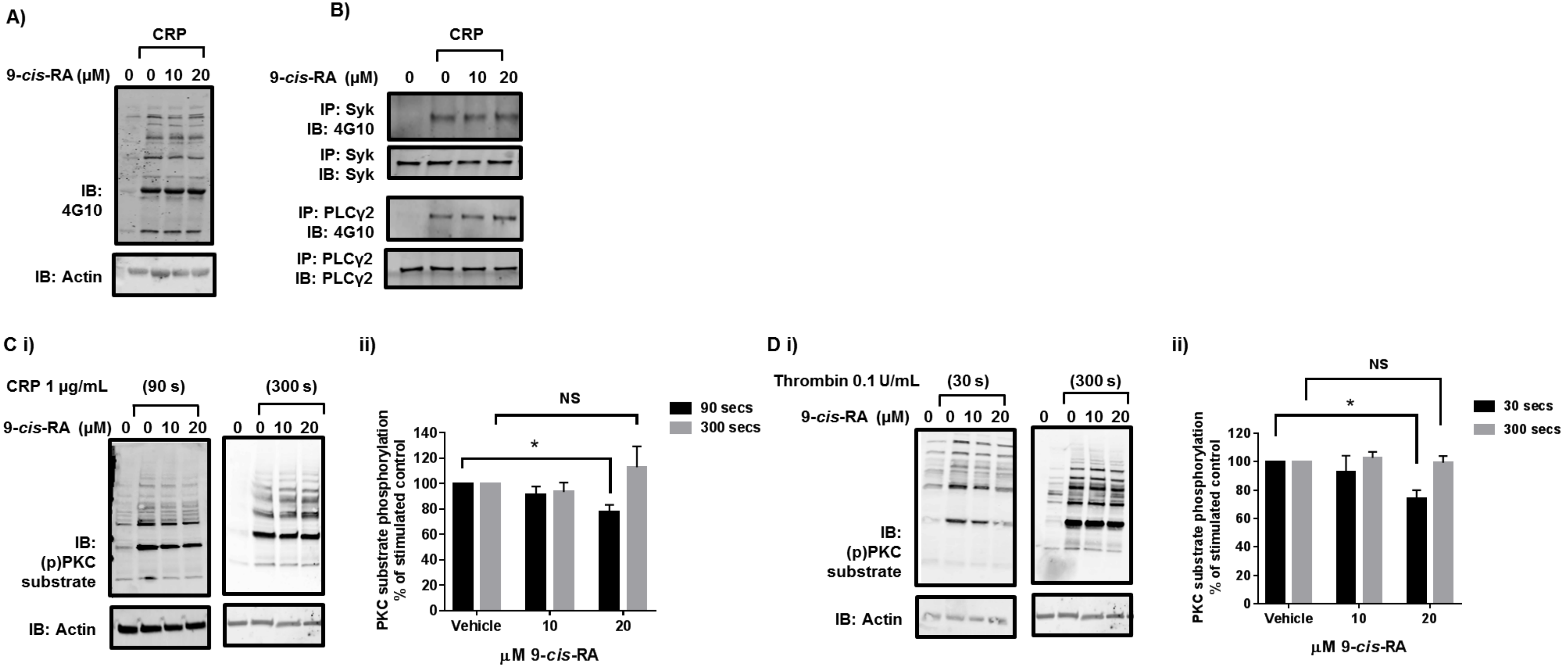

Supplemental Figure V. RXR ligands and PKA activity. Resting human washed platelets were treated with A) methoprene acid (10, 20 µM) or B) Docosahexaenoic acid (DSA) in the presence and absence of PKA inhibitor H89 (10 µM) for 10 minutes and samples tested for VASP S157 phosphorylation, a marker of PKA activity. PGI2 an activator of PKA activity was included as a positive control. Blotting samples were lysed in Laemmli sample buffer before separation by SDS PAGE gels and transfer onto PVDF membranes. Actin was used as a loading control. i) Representative blots are shown and ii) levels of phosphorylation were quantified and expressed as fold increase compared to vehicle control. Results are mean + S.E.M. for n≥3, * indicates p≤0.05 in comparison to vehicle controls.

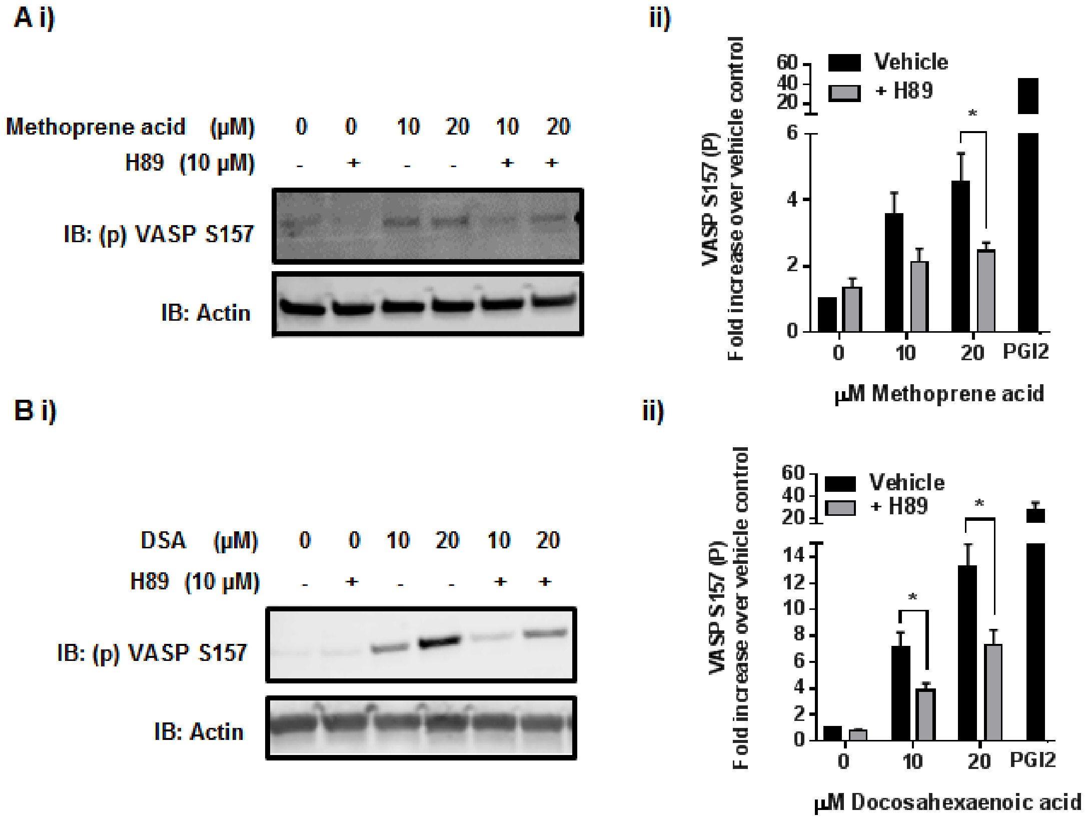

Supplemental Figure VI. 9-cis-RA dependent VASP S157 phosphorylation is not dependent on PKC or AKT activity. Resting human washed platelets were treated with 9-*cis*-RA (10, 20 µM) in the presence and absence of A) a PKC inhibitor GF109203X (GFX) (10 µM) B) an AKT inhibitor AKT inhibitor IV (AKTI IV) (5 µM) for 10 minutes and samples tested for VASP S157 phosphorylation, a marker of PKA activity. Blotting samples were lysed in Laemmli sample buffer before separation by SDS PAGE gels and transfer onto PVDF membranes. Actin was used as a loading control. i) Representative blots are shown and ii) levels of phosphorylation were quantified and expressed as fold increase compared to vehicle control. Results are mean + S.E.M. for n≥3, * indicates p≤0.05 in comparison to vehicle controls.

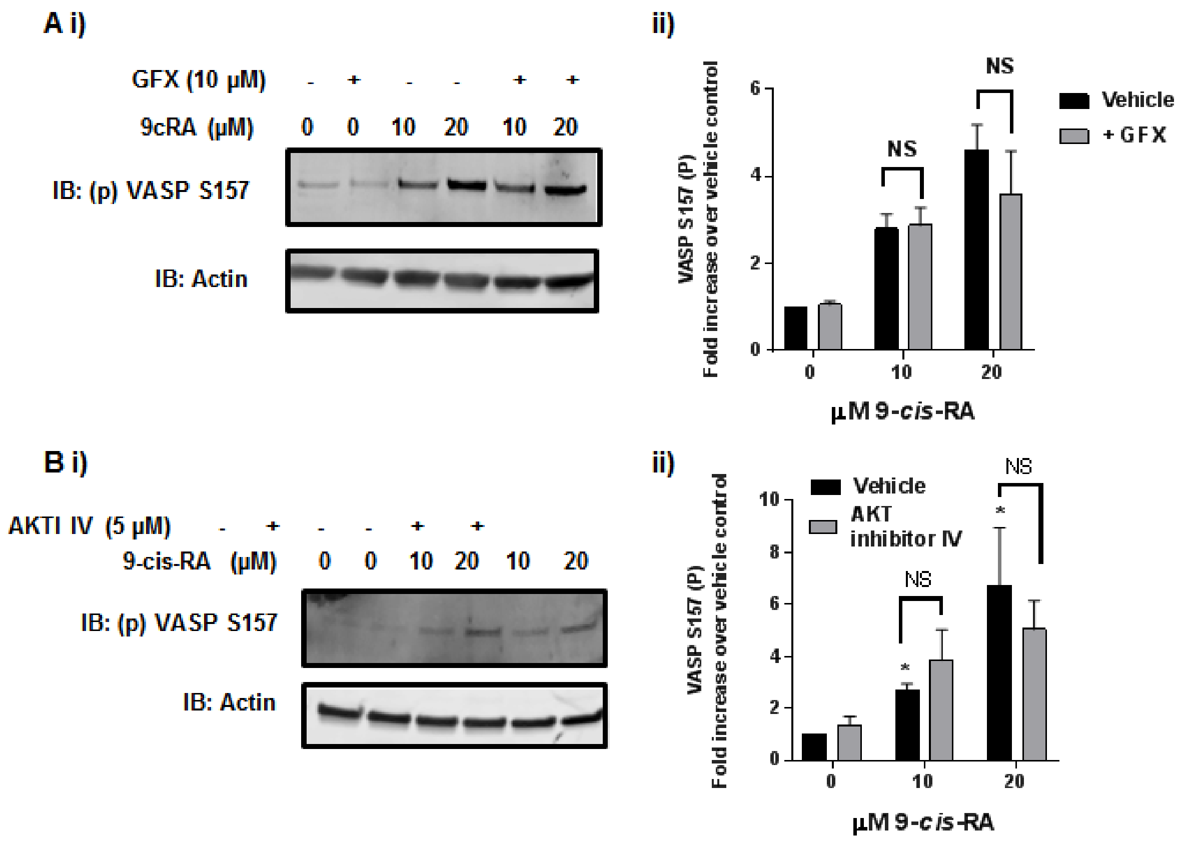

Supplemental Figure VII. RXR ligand dependent activation of PKA. Resting human washed platelets were treated with A,C) docosahexaenoic acid (10, 20 µM) or B,D) methoprene acid (10, 20 µM) for 10 minutes and in the presence and absence of A,B) phosphodiesterase inhibitor IBMX (1 mM) and cAMP levels measured using a cAMP ELISA kit (GE Healthcare as per manufacturers instructions). And C,D) an NFkB inhibitor, IKK inhibitor VII (5 µM) and samples tested for VASP S157 phosphorylation, a marker of PKA activity. Blotting samples were lysed in Laemmli sample buffer before separation by SDS PAGE gels and transfer onto PVDF membranes. Actin was used as a loading control. i) Representative blots are shown and ii) levels of phosphorylation were quantified and expressed as fold increase compared to vehicle control. Results are mean + S.E.M. for n≥3, * indicates p≤0.05 in comparison to vehicle controls.

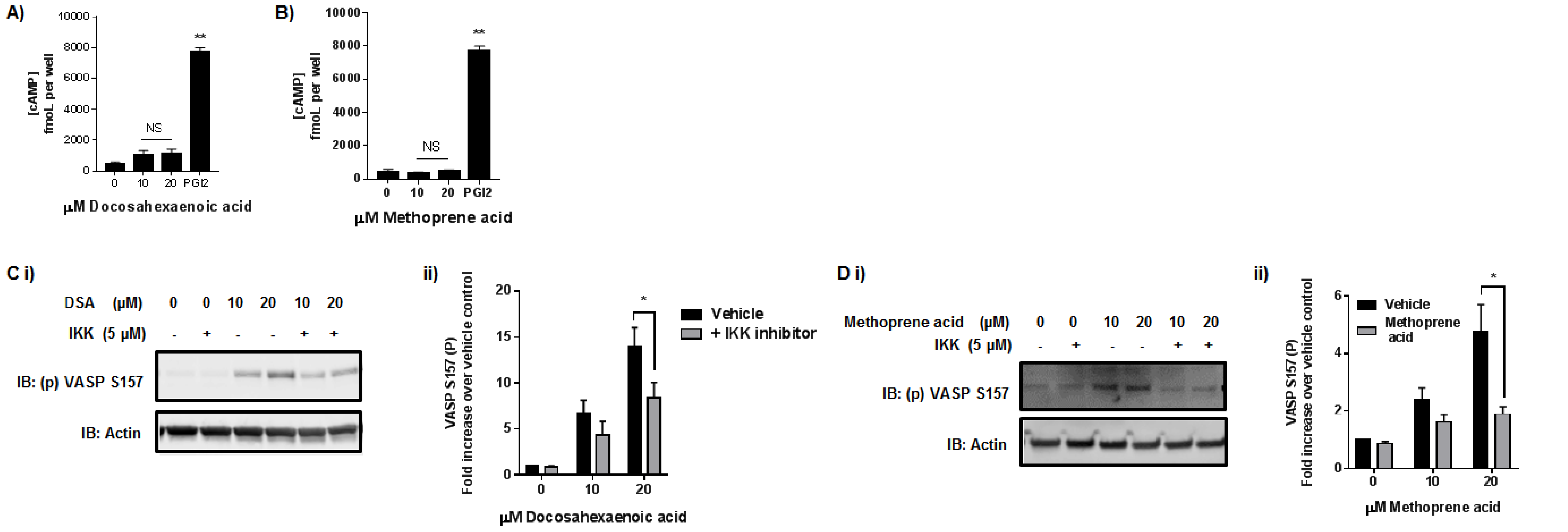

Supplemental Figure VIII. RXR ligand dependent activation of PKA via NFκB is not dependent on PPARs. Resting human washed platelets were treated with A) 15dPGJ2 (10, 20 µM) a PPARγ ligand or B) LG101506 (10 , 20 µM) a RXR:PPAR heterodimer modulator, for 10 minutes in the presence and absence of an NFkB inhibitor, IKK inhibitor VII (5 µM) and samples tested for VASP S157 phosphorylation, a marker of PKA activity. Blotting samples were lysed in Laemmli sample buffer before separation by SDS PAGE gels and transfer onto PVDF membranes. Actin was used as a loading control. i) Representative blots are shown and ii) levels of phosphorylation were quantified and expressed as fold increase compared to vehicle control. Results are mean + S.E.M. for n≥3, * indicates p≤0.05 in comparison to vehicle controls.

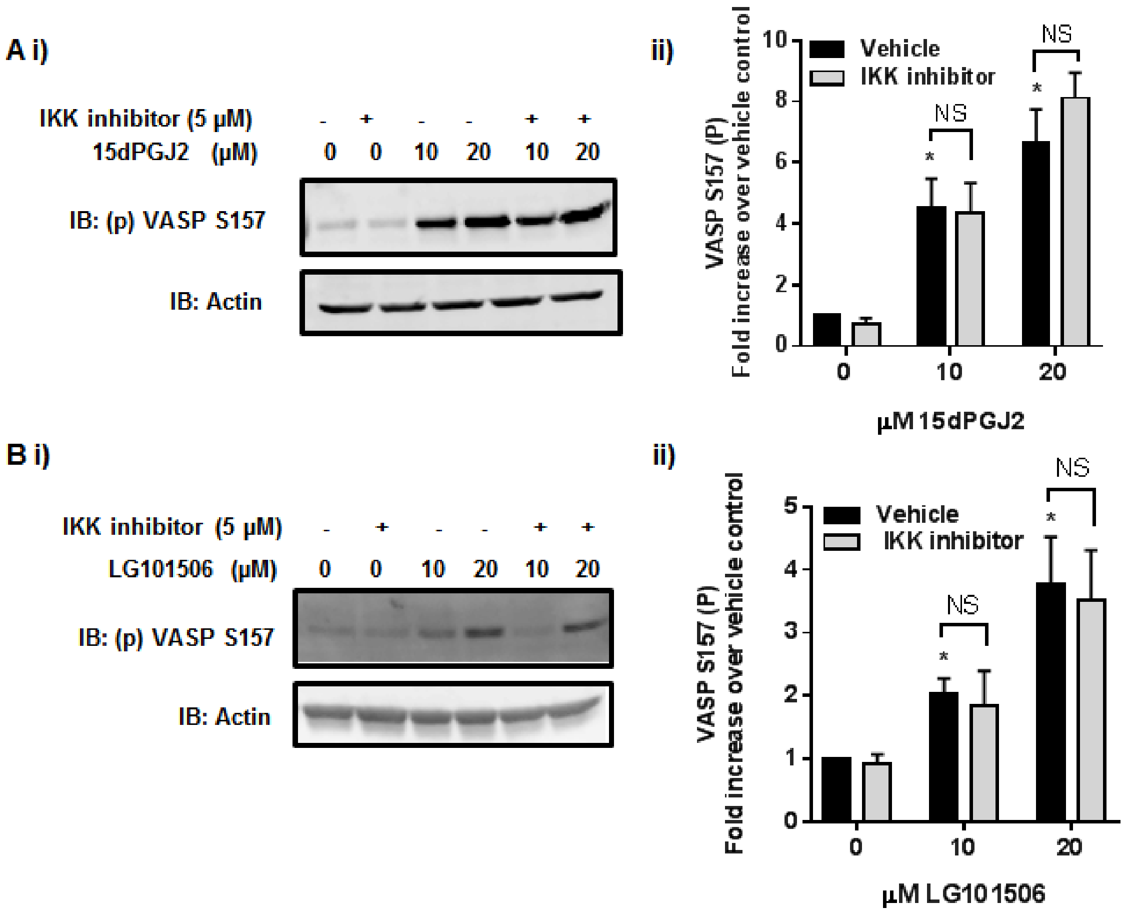

Supplement: Supplementary file 2 [file atv-37-812-s002.pdf]
