# Supplementary figures and images for: RXR Ligands Negatively Regulate Thrombosis and Hemostasis
Source: Arterioscler Thromb Vasc Biol. 2017 Mar 2;37(5):812–22. doi: 10.1161/ATVBAHA.117.309207 (PMC5405776; doi:10.1161/ATVBAHA.117.309207)

RXR ligands  
(9-*cis*-retinoic acid/vitamin A derivatives)

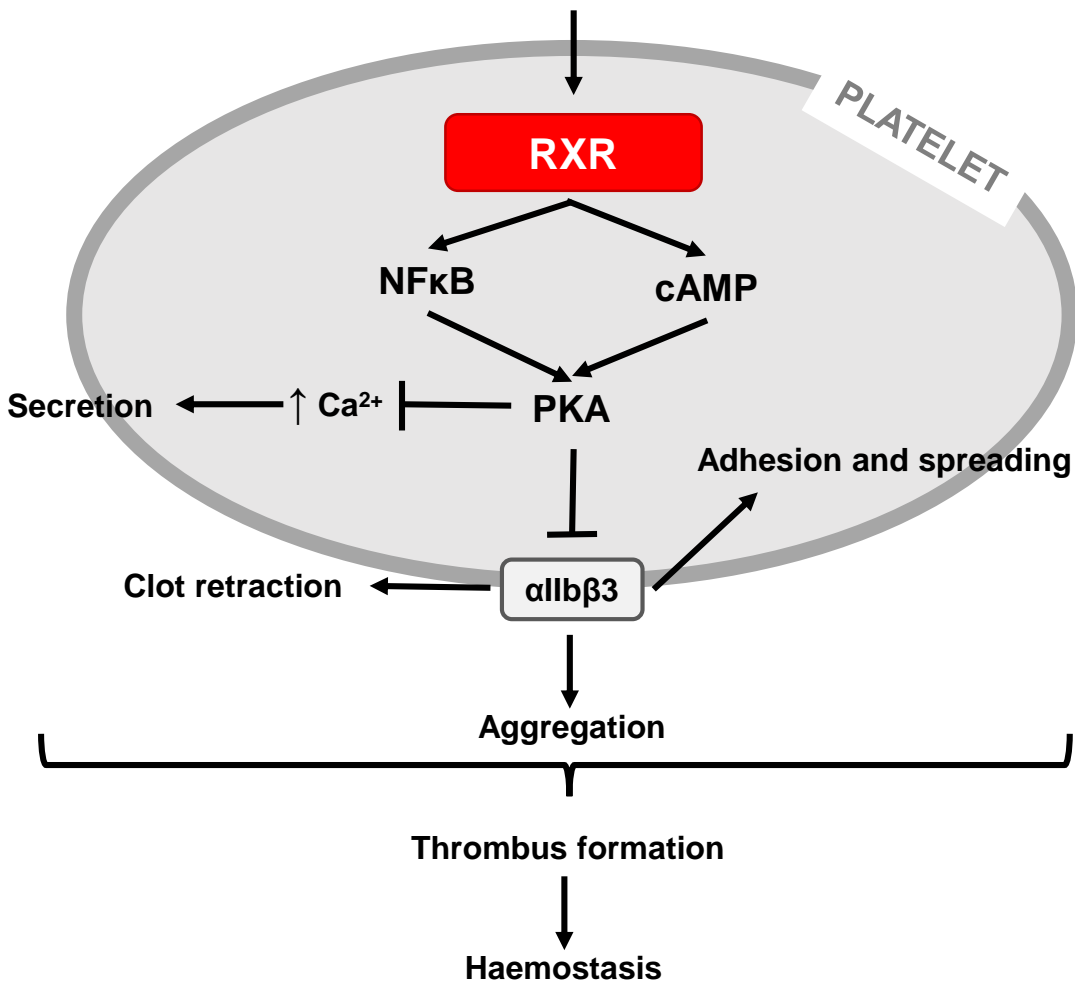

Supplement: Supplementary file 3 [file atv-37-812-s003.pdf]
